# Supplementary material for: Results from a maternal, newborn and child health program targeting pregnant, married adolescent girls in northern Nigeria
Source: PLOS Glob Public Health. 2026 Feb 2;6(2):e0004943. doi: 10.1371/journal.pgph.0004943 (PMC12863496; doi:10.1371/journal.pgph.0004943)
Supplement: S2 File — (DOCX) [file pgph.0004943.s002.docx]

S2 File. Human Centered Design Process

To address the gap adolescent mothers face in accessing maternal healthcare we sought to identify barriers that prevent married adolescent girls in northern Nigeria from attending ANC and to co-design an intervention to improve attendance and support healthy pregnancies. Our objective was to create an approach rooted in girls’ lived realities and responsive to the local context, while feasible to deliver at scale through the public health system

We used a human-centered design (HCD) process to develop the intervention. Human-centered design is a creative approach to problem solving that is rooted in empathy and collaboration. Our approach consisted of three phases: insight gathering, early or ‘rough’ prototyping, and small-scale pilot or ‘live’ prototyping. In each phase, we worked directly with adolescent girls and their influencers (husbands, mothers-in-law, community and religious leaders, and health care providers) to identify challenges and opportunities and get rapid feedback and suggestions on implementation approaches.

In the insight gathering phase, we spoke with 38 adolescent girls and young women and 89 additional community members to understand their experiences with pregnancy planning, identification, care and related topics. Alongside traditional research methods, we employed participatory techniques such as storytelling, journey mapping, role play, and card sorting. The entire research team, which included youth representatives, worked together to synthesize the responses and data into a series of insights. Prioritized insights included (1) limited planning among couples before pregnancy (2) restrictive norms around early ANC initiation (3) girls’ limited agency to seek maternal health care (4) concerns over the cost of ANC and (5) poor-quality or absent postpartum family planning discussions.

The team then identified opportunities linked to the insights. These were framed as ‘How Might We’ questions to provide a sense of openness and possibility. Relevant questions included (1) How might we create a natural opportunity for couples to discuss pregnancy timing and care before pregnancy happens? (2) How do we introduce an alternative ‘norm’ around the importance of ANC during the first trimester? (3) How might we give girls the self-efficacy and knowledge they need to confidently make decisions about what care they want during pregnancy? (4) How might we work with couples to plan for and prioritize costs of ANC? (5) How might we incorporate discussions around postpartum family planning as a routine component of ANC?

Next, the team brainstormed concepts that responded to the How Might We questions. During this process team members speculated about services, products, tools or other ideas that could capitalize on the opportunity identified. These ideas were reviewed, condensed, prioritized, and then turned into rough prototypes. Rough prototypes are tangible expressions of ideas that are easy and inexpensive to develop, and which can be shared with potential users for feedback.

Three different concepts were tested during rough prototyping.

1. ANC Gallery: This prototype was designed to help husbands see the value of antenatal care (ANC) services. It was aimed to showcase best-in-class examples of men supporting their wives to attend ANC, teach husbands about good ANC practices, and inspire action towards correct pregnancy care. Elements tested included a poster showcase, engagement with ANC experts, and follow-up to help men act on desires for a healthy pregnancy.
2. ANC Live: This prototype was designed to give pregnant girls and their mothers-in-law access to information on ANC through roleplay and discussion with experts. The sessions were delivered separately for girls and mothers-in-law. The goal was to transform mothers-in-law into advocates of ANC services and other healthy pregnancy behaviors and to equip young women with the information and confidence required to advocate for their own health needs.
3. ANC Journey Map: This prototype was designed to help girls have greater awareness of the changes happening to their bodies and the development of their babies during pregnancy. It was an engaging session with young women where they are shown a journey map that visually represents the changes month-by-month during pregnancy and highlights key moments for positive health behaviors, including ANC visits. The sessions were led by ANC experts such as government providers.

The prototypes were tested with 44 girls, 16 husbands, and 20 mothers-in-law in two health facilities in Kaduna state. The feedback provided during this period both furthered our understanding of the barriers and enablers of ANC care-seeking behavior, in general, and provided input into what did and did not work in the specific concepts tested. For example, through feedback on ANC Live girls helped us understand the tension they feel between exercising their own agency and the importance of showing respect to elders. What they most wanted was to create rapport and relate well with their supporters so there could be mutual agreement on the importance of attending ANC. A more specific lesson was that the ANC journey maps had too much content and were therefore hard for girls to understand. Importantly, this phase reinforced the importance of working with husbands to smooth girls’ access to ANC. Mothers-in-law, however, were identified as a lower priority audience given the logistical challenges of identifying and mobilizing this group. The element focusing on their involvement was therefore dropped in subsequent testing.

With this additional round of feedback and data the team refined the prototypes and developed a cohesive implement approach that combined the most effective elements of the original concepts. This resulted in an approach which closely mirrored the final implementation strategy. This intervention was then tested in a very small pilot known as ‘live prototyping’ for a period of approximately three months. In this phase, the intervention ran continuously (i.e. girls and husbands were continuously recruited into the program) and qualitative and quantitative data was collected to assess how well the program operated and if it was likely to achieve the desired aims. Lessons from this period informed the final intervention design for the pilot, which was then tested through the effectiveness evaluation.

The final versions of the ANC Gallery posters are available [here](https://a360learninghub.org/resource/mma-implementation-asset-anc-gallery-posters/). The final version of the ANC Journey Map are available [here](https://a360learninghub.org/resource/mma-implementation-asset-anc-journey-brochure-by-trimester/).
