# Supplementary material for: Results from a maternal, newborn and child health program targeting pregnant, married adolescent girls in northern Nigeria
Source: PLOS Glob Public Health. 2026 Feb 2;6(2):e0004943. doi: 10.1371/journal.pgph.0004943 (PMC12863496; doi:10.1371/journal.pgph.0004943)
Supplement: Attachment — Submitted filename: Response_A360_MNCH.docx [file pgph.0004943.s005.docx]

**Response to Reviewers**

**Journal Requirements:**

1. Please include a complete copy of PLOS’ questionnaire on inclusivity in global research in your revised manuscript. Our policy for research in this area aims to improve transparency in the reporting of research performed outside of researchers’ own country or community. The policy applies to researchers who have travelled to a different country to conduct research, research with Indigenous populations or their lands, and research on cultural artefacts. The questionnaire can also be requested at the journal’s discretion for any other submissions, even if these conditions are not met. Please find more information on the policy and a link to download a blank copy of the questionnaire here: <https://journals.plos.org/globalpublichealth/s/best-practices-in-research-reporting>. Please upload a completed version of your questionnaire as Supporting Information when you resubmit your manuscript.

2. Please amend your detailed Financial Disclosure statement. This is published with the article. It must therefore be completed in full sentences and contain the exact wording you wish to be published.

i. State the initials, alongside each funding source, of each author to receive each grant.
ii. State what role the funders took in the study. If the funders had no role in your study, please state: “The funders had no role in study design, data collection and analysis, decision to publish, or preparation of the manuscript.” Updated.

3. Please ensure that your Ethics Statement is available in its entirety at the beginning of your Methods section, under a subheading 'Ethics Statement'. It must include:

1) The name(s) of the Institutional Review Board(s) or Ethics Committee(s)

2) The approval number(s), or a statement that approval was granted by the named board(s)

3) (for human participants/donors) - A statement that formal consent was obtained (must state whether verbal/written) OR the reason consent was not obtained (e.g. anonymity).

NOTE: If child participants, the statement must declare that formal consent was obtained from the parent/guardian.

Updated.

4. Please provide separate figure files in .tif or .eps format. N/A. No figures.

For more information about figure files please see our guidelines:

<https://journals.plos.org/globalpublichealth/s/figures> 
<https://journals.plos.org/globalpublichealth/s/figures#loc-file-requirements>

5. We have noticed that you have uploaded Supporting Information files, but you have not included a list of legends. Please add a full list of legends for your Supporting Information files after the references list. Created a new sub-heading after references listing the Supporting Information files.

6. Please note that your Data Availability Statement is currently missing the repository name and/or the DOI/accession number of each dataset OR a direct link to access each database. If your manuscript is accepted for publication, you will be asked to provide these details on a very short timeline. We therefore suggest that you provide this information now, though we will not hold up the peer review process if you are unable.

There was no Data Availability Statement in the original submission of the article, so I have added this section and provided the license information, title, and DOI.

While revising your submission, we strongly recommend that you use PLOS’s NAAS tool (<https://ngplosjournals.pagemajik.ai/artanalysis>) to test your figure files. NAAS can convert your figure files to the TIFF file type and meet basic requirements (such as print size, resolution), or provide you with a report on issues that do not meet our requirements and that NAAS cannot fix. N/A – no figures

After uploading your figures to PLOS’s NAAS tool - <https://ngplosjournals.pagemajik.ai/artanalysis>, NAAS will process the files provided and display the results in the "Uploaded Files" section of the page as the processing is complete. If the uploaded figures meet our requirements (or NAAS is able to fix the files to meet our requirements), the figure will be marked as "fixed" above. If NAAS is unable to fix the files, a red "failed" label will appear above. When NAAS has confirmed that the figure files meet our requirements, please download the file via the download option, and include these NAAS processed figure files when submitting your revised manuscript. N/A – no figures

**Response to Reviewers**

Thank you to the reviewers and editors for their substantial comments. We have listed below each comment and our response. We have also made tracked changes in the document in the relevant places.

**Reviewer 1.**

Comment 1: This paper summarizes results from a longitudinal quasi-experimental study evaluating the effectiveness of a maternal, newborn and child health (MNCH) intervention on pregnancy and postpartum outcomes among married adolescent girls in northern Nigeria. The paper is well written from the statistical perspective. However, there are some clarifications needed. Although comparative, the study is nonrandomized using some LGA sites as the intervention sites and others as the control or comparison. The study appears large enough.

Comment 2: Since treatment had been pre-assigned did the investigators consider any matching such as propensity score matching or other method to assure further comparability of the samples to each other? With a 3:1 assignment this may be challenging, but can be done.

- Response 2: Thank you for this comment. We did consider exploring propensity score matching, but were worried about the reduction in sample size. Matching with replacement as suggested could help mitigate some of that reduction. We decided instead to generate propensity scores and weight the regression models by them (inverse probability weights). As you will see, the results remained primarily the same with some small changes to coefficients.

Comment 3: The sample size calculation was reasonable. However, the dropout or loss rate for each group is higher than 10%. Were any retention or sample augmentation strategies implemented into the design? This should be added as a limitation in the discussion section.

- Response 3: Thank you for this comment. The sample size was calculated for the larger sample which included not only pregnant girls, but girls at risk for pregnancy. This paper is a sub-group analysis for pregnant girls. The sample size calculation for the entire sample was augmented by 10% to account for loss-to-follow up. However, we did not add additional sample augmentation specifically for the sub-analysis of pregnant women. We have noted this in the limitations section on page 26 in the manuscript.

Comment 4: The data analysis is routine in this setting using between group t-tests and chi-square tests of comparison), and multivariate linear and modified Poisson regression. In the regression, please explain the controlling for the LGA. The quantitation structure for this variable is not clear.

- Response 4: Local Government Areas (LGA) could be thought of as a county in the U.S. We controlled for LGA to account for potential geographic differences that could influence the outcome-exposure relationship. Controlling for LGA (“location”) enables us to remove the effects of location (any the different characteristics of each location) to obtain a more accurate and unbiased estimate between the outcome and main exposure variable.
  - Data was collected in five intervention Local Government Areas (LGAs) (Zaria and Sabon Gari in Kaduna state and Kiyawa, Ringim and Taura in Jigawa state) and in four comparison LGAs (Igabi and Chikun in Kaduna and, Auyo and Kafin Hausa in Jigawa). Selection of LGAs was conducted using purposive sampling, selecting LGAs already implementing MMA for the intervention group and following recommendations by the State Ministry of Health officials. There were 30 PHCs for the intervention group allocated equally to intervention LGAs for a total of six PHCs per LGA. There were 25 comparison PHCs (15 in Kaduna and 10 in Jigawa allocated to the two states based on the cumulative program performance for the SRH outcomes for the six-months’ period prior to study implementation).

**Reviewer 2.**

Comment 1: The authors provide sufficient justification for conducting this study in Northern Nigeria, and make a good case for the manuscripts contribution to the literature on SRH care for adolescents in similar settings. Kudos to the team.

- Response 1: Thank you for this comment.

Comment 2: Line 164: The authors highlight the importance of recognizing adolescents as partners in designing health programs. Given that this is an important component of the intervention, I suggest the authors include more information about the HCD process, as a supplement.

- Response 2: Thank you for this point. Yes, we agree. We have added more about the HCD process in a supplement document (S2. Text) and have referred readers to that document on Page 7.

Comment 3: Line 250: I suggest the authors provide more information on the criteria/method used in selecting the intervention and comparison sites within the study areas

- Response 3: Data was collected in five intervention Local Government Areas (LGAs) (Zaria and Sabon Gari in Kaduna state and Kiyawa, Ringim and Taura in Jigawa state) and in four comparison LGAs (Igabi and Chikun in Kaduna and, Auyo and Kafin Hausa in Jigawa). Selection of LGAs was conducted using purposive sampling, selecting LGAs already implementing MMA for the intervention group and following recommendations by the State Ministry of Health officials. here were 30 PHCs for the intervention group allocated equally to intervention LGAs for a total of six PHCs per LGA. There were 25 comparison PHCs (15 in Kaduna and 10 in Jigawa allocated to the two states based on the cumulative program performance for the SRH outcomes for the six-months’ period prior to study implementation). Participants in the intervention facilities received MMA enhanced with the MNCH intervention components, participants in the comparison group received MMA and standard of care MNCH services based on MOH guidelines.  In both cases selection of LGAs was purposive ensuring that intervention and comparison areas were separated by substantial geographic distance to reduce spillover of effects. We have added a sentence on page 8-9 for clarification.

Comment 4: Line 257/258: The authors indicate their interest in measuring incremental effectiveness. I suggest the authors include some information on the outcomes of the MMA component alone, to provide some bases/context for understanding the intended addtional effect of the MNCH component.

- Response 4: We have added in additional information in the background section on the outcomes of MMA as well as tried to make the intended additional effect more clear in the methods section.

Comment 5: Line 259: It is unclear who the target population is for the MNCH component of the intervention. Do the authors mean that adolescent girls who were already recruited for the MMA component were then recruited again for the MNCH component when they got pregnant at the 3-month follow-up, or did the authors provide both components of the intervention to a cohort of girls who met the eligibility criteria in the study areas?

- Response 5: Thank you for this comment. We have tried to make this clearer in the manuscript. The target population for this MNCH component was adolescent girls who lived in the catchment area and met eligibility criteria. The MMA and MNCH component were provided to a cohort of girls who met eligibility criteria in the intervention area. In the control areas, only the MMA component was provided.

Comment 6: Line 277: The sample size was estimated to detect an effect size between baseline and the 3-month follow-up. However, the authors indicate in Line 230 that this paper presents results from the post-partum survey. It is unclear how if the sample size calculated is indeed powered enough to detect effects post-partum. Some food for thought and clarity by the authors.

- Response 6: The sample size for this study was calculated to detect the effect size between baseline and 3-month follow-up as noted by the reviewer. We have noted in the limitations section that power analysis was not calculated for the sub-analysis for pregnant women only.

Comment 7: Line 285: Since the postpartum survey was conducted only for participants who were pregnant at baseline or at the 3-month follow-up, it will be important to present the baseline characteristics of these participants (e.g gestation of pregnancy). If possible, the authors could stratify their results by participants pregnant at baseline and those pregnant at the 3-month follow-up. I suspect metrics such as number of ANC visits and prevalence of ANC/PNC attendance might differ for these two groups, which provides an important indicator of the timing of the proposed intervention.

- Response 7: Thank you for this comment. In the entire study sample (not just those in the postpartum sample), 74.23% of participants were pregnant at baseline. There were no new pregnancies at 3-month follow-up. Among those who were not pregnant at baseline so we cannot do the suggested sub-analysis.

Comment 8: I suggest the authors provide information on the fidelity/dose/reach of the all, aspects of the MNCH component in both the intervention and comparison groups. At present, this is assumed to be the same.

- Response 8: Thank you for this comment. While both groups participated in programming, only the intervention group received the MNCH component. The dosage of this was one, 90-min session focused on education about ANC, introduction to ANC providers, and reflection activities about individual motivations for attending ANC and asking for support from family members. Participants in the intervention group also received referrals to ANC and follow-up in the form of phone calls or home visits if they did not attend. In intervention sites, husbands also received information about the importance and value of ANC. None of this was available in comparison sites.

Comment 9: Line 327: Given that education was identified as an key determinant of access to care, and the relatively large diffence in education between the intervention and comparison groups, it might be useful to consider a sub-group analysis by educational status (education vs no education)

Response 9: Thank you for this comment. We have examined education as a categorical variable. But, to look at potential differences by education, we created a binary version, added in a moderation term for education/no education to test for potential differences by education level. Given the small sample size of no education, the interaction effect was not SS. Furthermore, this was of those who reported the highest level of schooling note those currently in school.

Comment 10: Line 338: If the mean gestational age at first ANC was 4.9 months, does this mean that at baseline (and at 3-months follow-up), all participants had not attended ANC? I think it is important to present a table of demographics for all participants (intervention and comparison groups) at baseline to provide more analytical context for the study

- Response 10: Thank you for this comment. Eligibility criteria included not having started ANC at baseline (page 9, line 267). Given that all girls in the postpartum sample were pregnant at baseline, we do not see the need for another table presenting the larger study sample.

Comment 11: Table 3: Given that LGA remains significant in ANC/PNC attendance (prevalence and number of ANC visits), and the fact that distance to facility of care was highlighed as one of the barriers to accessibility, I suggest the authors add this to their discussion and suggest ways that further studies might account for this salient factor.

- Response 11: Thanks for this comment. We have added in reflections on the influence of LGA into the discussion section on Page 22.
